# A plant-specific DYRK kinase DYRKP coordinates cell morphology in *Marchantia polymorpha*

**DOI:** 10.1007/s10265-021-01345-w

**Published:** 2021-09-21

**Authors:** Tomoyuki Furuya, Haruka Shinkawa, Masataka Kajikawa, Ryuichi Nishihama, Takayuki Kohchi, Hideya Fukuzawa, Hirokazu Tsukaya

**Affiliations:** 1 Graduate School of Science, The University of Tokyo, Tokyo, 113- 0033 Japan; 2 Graduate School of Science, Kobe University, Kobe, 657-8501 Japan; 3 Graduate School of Biostudies, Kyoto University, Kyoto, 606-8502 Japan; 4 Present Address: Research Institute for Bioresources and Biotechnology, Ishikawa Prefectural University, Ishikawa, 921-8836 Japan; 5 Present Address: Faculty of Biology-Oriented Science and Technology, Kindai University, Wakayama, 649-6493 Japan; 6 Present Address: Faculty of Science and Technology, Tokyo University of Science, Chiba, 278- 8510 Japan

**Keywords:** Cell shape, Liverwort, Morphogenesis, Protein kinase

## Abstract

Dual-specificity tyrosine phosphorylation-regulated kinases (DYRKs) are activated via the auto-phosphorylation of conserved tyrosine residues in their activation loop during protein translation, and they then phosphorylate serine/threonine residues on substrates. The DYRK family is widely conserved in eukaryotes and is composed of six subgroups. In plant lineages, DYRK homologs are classified into four subgroups, DYRK2s, yet another kinase1s, pre-mRNA processing factor 4 kinases, and DYRKPs. Only the DYRKP subgroup is plant-specific and has been identified in a wide array of plant lineages, including land plants and green algae. It has been suggested that in *Arabidopsis thaliana* DYRKPs are involved in the regulation of centripetal nuclear positioning induced by dark light conditions. However, the molecular functions, such as kinase activity and the developmental and physiological roles of DYRKPs are poorly understood. Here, we focused on a sole DYRKP ortholog in the model bryophyte, *Marchantia polymorpha*, MpDYRKP. MpDYRKP has a highly conserved kinase domain located in the C-terminal region and shares common sequence motifs in the N-terminal region with other DYRKP members. To identify the roles of MpDYRKP in *M. polymorpha*, we generated loss-of-function Mp*dyrkp* mutants via genome editing. Mp*dyrkp* mutants exhibited abnormal, shrunken morphologies with less flattening in their vegetative plant bodies, thalli, and male reproductive organs, antheridial receptacles. The surfaces of the thalli in the Mp*dyrkp* mutants appeared uneven and disordered. Moreover, their epidermal cells were drastically altered to a narrower shape when compared to the wild type. These results suggest that MpDYRKP acts as a morphological regulator, which contributes to orderly tissue morphogenesis via the regulation of cell shape.

**Supplementary Information:**

The online version contains supplementary material available at 10.1007/s10265-021-01345-w.

## Introduction

The dual-specificity tyrosine (Y) phosphorylation-regulated kinase (DYRK) family belongs to the larger Cdk2, MAPK, GSK3, CLK and related kinase family (CMGC), that has been identified in a wide range of animals, plants, fungi, and protists (Aranda et al. 2011; Manning et al. 2002; Varjosalo et al. 2013). Generally, DYRKs are activated via the auto-phosphorylation of a conserved Y residue in the activation loop during protein translation and they then phosphorylate serine/threonine (S/T) residues on substrates. (Lochhead et al. 2005; Soppa and Becker 2015). The DYRK family is composed of six subgroups: DYRK1s, DYRK2s, yet another kinase1s (Yak1s), pre-mRNA processing factor 4 kinases (PRP4Ks), homeodomain-interacting protein kinases (HIPKs), and plant-specific DYRKs (DYRKPs) (Kajikawa et al. 2015). DYRKs in yeast and mammals are well known as key regulators of the cell cycle and differentiation (Aranda et al. 2011; Becker 2012; Soppa and Becker 2015). Yak1 in *Saccharomyces cerevisiae*, which is a founding member of the DYRK family, is a negative regulator of proliferation under nutritional stress (Garret and Broach 1989). Mammalian DYRK1A, which is well known as a candidate gene for involvement in Down syndrome, functions in neuronal differentiation (Gwack et al. 2006; Kurabayashi and Sanada 2013). In addition, DYRK2 plays important roles in cell cycle and apoptosis regulation (Nihira and Yoshida 2015; Taira et al. 2007, 2012).

There are only four DYRK subgroups in plant lineages, including land plants and green algae, and these include DYRK2, Yak1, PRP4K, and DYRKP. Triacylglycerol accumulation regulator1 (TAR1), a homolog of the Yak subgroup in *Chlamydomonas reinhardtii*, was identified as the gene responsible for the low triacylglycerol (TAG) accumulation mutant that is known to occur in sulfur and nitrogen deficient conditions (Kajikawa et al. 2015). Under nitrogen-deficient conditions, TAR1 also regulates the growth and degradation of chlorophyll and photosynthesis-related proteins. The loss-of-function mutant for *Arabidopsis thaliana* Yak homolog, AtYAK1, showed less sensitivity to the plant hormone abscisic acid (ABA) in the context of seed germination, seedling growth, and stomatal closure, suggesting that AtYAK1 positively regulates the ABA signaling transduction pathway (Kim et al. 2016). In addition, AtYAK1 was also found to interact with LIGHT-REGULATED WD1 (LWD1) and LWD2, which are key regulators of the circadian period (Huang et al. 2017). Indeed, *atyak1* mutants have a longer circadian period length and delayed flowering phenotype than the wild type (Huang et al. 2017). Moreover, AtYAK1 functions downstream of TARGET OF RAPAMAYCIN (TOR) kinase, which is involved in the regulation of meristem activity and cell differentiation during root development (Barrada et al. 2019; Forzani et al. 2019; Xiong et al. 2013).

Unlike the other subgroups, the DYRKP subgroup is only found in plant genomes (Kajikawa et al. 2015; Schulz-Raffelt et al. 2016). In *A. thaliana*, four DYRKPs (DYRKP-1, DYRKP-2 A, DYRKP-2B, and DYRKP-3) are encoded in the genome (Iwabuchi et al. 2019). These DYRKPs were shown to interact with ANGSTIFOLIA (AN) in the yeast two-hybrid system, and it is suggested that this complex contributes to dark-induced centripetal nuclear positioning via the alignment of actin filaments (Iwabuchi et al. 2019). In addition, DYRKP in *C. reinhardtii* negatively regulates the accumulation of starch and oil synthesis in low energy status conditions (Schulz-Raffelt et al. 2016). However, the functions of DYRKPs in other contexts, including their morphological, developmental, and evolutionary aspects, requires further elucidation.

Here, we focused on the liverwort *Marchantia polymorpha*, which is part of the bryophyte (Bowman et al. 2017). *M. polymorpha* is a well-established model plant with various experimental advantages such as a short life cycle, ease of culturing and crossing, a low level of genetic redundancy, and available genetic information and gene manipulation systems (Ishizaki et al. 2016; Kohchi et al. 2021; Shimamura 2016). Indeed, *M. polymorpha* has a single DYRKP ortholog, MpDYRKP. We generated genome-edited mutants, Mp*dyrkp*, which exhibited defects in their thallus morphology as they showed shrinking and less flattening. Moreover, epidermal cells in Mp*dyrkp* mutants were drastically altered to narrower shapes compared to the wild type. Our findings provide novel knowledge regarding the role of DYRKPs in terms of morphology.

## Materials and methods

### Plant materials and culture conditions

The *M. polymorpha* male accession Takaragaike-1 (Tak-1) was used as the wild-type line (Ishizaki et al. 2008; Okada et al. 2000). Wild-type and transgenic plants were cultured on half-strength Gamborg’s B5 medium (Gamborg et al. 1968) containing 1 % (w/v) agar with or without 1 % (w/v) sucrose or soil under 50–60 µmol m^−2^ s^−1^ continuous white light at 22 °C. To induce the reproductive phase, gemmalings were cultured on soil under continuous 50–60 µmol m^−2^ s^−1^ white light supplemented with 20–40 µmol m^−2^ s^−1^ far-red light (730 nm) irradiation (VBL-T600-1, Valore).

### Construction of transgenic plants

The MpDYRKP targeting vectors used for genome editing with the CRISPR/Cas9 system were constructed according to Sugano et al. (2018). MpDYRKP-gRNA-F1 5′-CTC GGT CCT ATT TTC AGG GGC A-3′ and MpDYRKP-gRNA-R1 5′-AAA CTG CCC CTG AAA ATA GGA C-3′ for guide RNA-1, and MpDYRKP-gRNA-F2 5′-CTC GAT CGT ACA CAG GAA AAA C-3′ and MpDYRKP-gRNA-R2 5′-AAA CGT TTT TCC TGT GTA CGA T-3′ for guide RNA-2 were incubated together at 95°C for 5 min, then cooled to 85 °C at a rate of − 2 °C s^−1^, held at 85 °C for 1 min, the further cooled to 25 °C at − 0.2 °C s^−1^ to enable annealing. The resultant annealed oligonucleotides containing the target sequence were inserted into the *Bsa*I site of pMpGE_En03 to construct the entry vector. The sequence between attL1 and attL2 within the entry vector was inserted into the binary vector pMpGE010 by the LR reaction using LR clonase II (Life Technologies). The vector used for the complementation analysis was constructed based on the method of Ishizaki et al. (2015). The genomic sequences of the promoter region (5-kb upstream of the start codon) and terminator region (1-kb downstream of the stop codon) of Mp*DYRKP* were amplified from the Tak-1 genomic DNA using the following primers: MpDYRKPpro-F 5′-TGG ATC CGG TAC CGA ATT CGG ACT TGG AAC ATC TAG C-3′ and MpDYRKPpro-R, 5′-CGA GTG CGG CCG CGA ATT GGC TGG ATA TGA TTG AGA TCA C-3′ for the promoter, and MpDYRKPterm-NotI-F 5′-TAT CTC CGC GCG GCC GTG AGA GTT ATC TTT CAA TC-3′ and MpDYRKPterm-NotI-R1 5′-ATC TCG AGT GCG GCC TGT GGT TTA CCA TAT AAT TC-3′’ for the terminator. The stop codon in the MpDYRKPterm-NotI-F primer is underlined in the above sequence. The longest Mp*DYRKP* coding sequence corresponding to the splicing variant Mp3g19940.1, was amplified from Tak-1 thalli cDNA using the following primers: MpDYRKPORF-NotI-F 5′-ATC CAG CCA ATT CGC GGC CAT GGC GGA TTC GGT CAA TGC C-3′ and MpDYRKPORF-NotI-R 5′-AGA TAT CTC GAG TGC GGC CGC GCG GAG ATA GGT TCA TAT GG-3′. The Mp*DYRKP* promoter region (hereafter _*pro*_Mp*DYRKP*) was cloned into pENTR1A digested with *Eco*RI using the SLiCE method (Motohashi 2015). Then, the MpDYRKP ORF was inserted into the *Not*I site of pENTR1A-_*pro*_Mp*DRYKP.* During this cloning, the *Not*1 site on the 3′ side of the ORF was preserved, while the *Not*1 site on the 5′ side of the ORF was not. Finally, the Mp*DYRKP* terminator region (hereafter Mp*DYRKP*term) was inserted into the *Not*I site of the pENTR1A-_*pro*_Mp*DYRKP*:Mp*DYRKP* ORF plasmid. The _*pro*_Mp*DYRKP*:Mp*DYRKP* ORF:Mp*DYRKP*term region between attL1 and attL2 within the resultant entry plasmid was inserted into the destination vector pMpGWB301 (Ishizaki et al. 2015) by LR reaction using LR clonase II to generate the _*pro*_Mp*DYRKP*:Mp*DYRKP* construct. To confirm the introduction of the _*pro*_Mp*DYRKP*:Mp*DYRKP* construct in the transformants, the primers, MpDYRKPORFE2-F1 5′-GCA AGG AGG CTT AGT GGA GGT A-3′ and MpDYRKPORFE2-R1 5′-TCT CCA CTT CCT CGT CAT CAT C-3′ were used for PCR screening.

A citrine fusion construct, _*pro*_Mp*DYRKP*:Mp*DYRKP*-*Citrine* was constructed as previously reported (Ishizaki et al. 2015). The constructed entry plasmid pENTR1A-_*pro*_Mp*DYRKP*:Mp*DYRKP* ORF, which did not have a stop codon, was used for LR cloning with the destination vector pMpGWB307 (Ishizaki et al. 2015).

The thallus transformation with the CRISPR/Cas9 system and complementation constructs were described previously (Kubota et al. 2013). Thalli from Mp*dyrkp-1*^*ge*^ and *Agrobacterium* containing the pMpGWB301-_*pro*_Mp*DYRKP*:Mp*DYRKP* vector or the pMpGWB307-_*pro*_Mp*DYRKP*:Mp*DYRKP-Citrine* vector were used for the transformation. Transformants were selected on half-strength B5 agar medium containing 1 % (w/v) agar, 0.5 µM chlorosulfuron, and 100 mg L^−1^ cefotaxime (Claforan; Sanofi-Aventis).

### Phylogenetic analysis

A phylogenetic tree for the DYRKs was constructed by comparing the amino acid sequences of their kinase domains. The tree was generated using MEGA X (version 10.1, https://www.megasoftware.net/; Kumar et al. 2018) with the maximum likelihood algorithm.

### Confocal microscopy

For the observation of apical notches, 3-day-old plants after gemma germination were fixed in PFA fixative solution [4% (w/v) paraformaldehyde, and 0.05% (v/v) Triton X-100 in Phosphate buffered saline (PBS)]. Samples were degassed by vacuum infiltration and incubated for 1 h at room temperature with gentle shaking. Fixed samples were rinsed with 0.05% (v/v) Triton X-100 in PBS and cleared in the ClearSee solution [10% (w/v) xylitol, 15% (w/v) sodium deoxycholate, and 25% (w/v) urea] with 0.02% (v/v) SCRI Renaissance 2200 (Renaissance Chemicals, Selby, UK) (Kurihara et al. 2015). After replacing with the ClearSee solution, samples were kept for two additional days or more. Samples were mounted on glass slides with the ClearSee solution for microscopic obervations. SCRI Renaissance 2200 fluorescence was visualized using the confocal laser scanning microscope (Olympus FLUOVIEW FV1000, Tokyo, Japan) at excitation and detection wavelengths of 405 and 425–460 nm, respectively. Images of plant tissues expressing the MpDYRKP-Citrine fusion protein were captured using a Leica TCS SP8 microscope (Leica Microsystems). A 488-nm argon laser was used for excitation, and fluorescence detected between 500 and 550 nm and 705–765 nm was from Citrine and chlorophyll, respectively.

### 5-Ethynyl-2’-deoxyuridine (EdU) staining

To visualize S-phase cells, the Click-iT EdU Imaging Kit (Life Technologies) was used. EdU staining was performed as described previously (Furuya et al. 2018; Naramoto et al. 2019; Nishihama et al. 2015), with a slight modification. Three-day-old plants after gemma germination were soaked in half-strength Gamborg’s B5 medium with 10 µM EdU at 22 °C for 1 h under continuous white light. Thalli were fixed in FAA [50 % (v/v) ethyl alcohol, 2.5 % (w/v) glacial acetic acid, and 2.5 % (w/v) formalin] and were degassed by vacuum infiltration. EdU in the fixed samples were detected with Alexa Fluor 555-azide following the manufacturer’s protocol. For clearing, samples were treated with the ClearSee solution for a few days. Samples were mounted on glass slides with the ClearSee solution; Alexa Fluor555 fluorescence was visualized using the confocal laser scanning microscope (Olympus FLUOVIEW FV1000, Tokyo, Japan) at excitation and detection wavelengths of 559 and 570–670 nm, respectively. Z-projection images were created using ImageJ software.

### Scanning electron microscopy

Thalli fixed in FAA were dehydrated using a graded ethanol series and isoamyl acetate, and dried using a JCPD-5 critical point dryer (JEOL, Tokyo, Japan). The samples were mounted using carbon tape and coated with platinum using a JEC-3000FC sputter-coater (JEOL). Images of epidermal cell surfaces were captured using a JSM-6510LV scanning electron microscope (JEOL, Tokyo, Japan). Epidermal cell margins were traced using Photoshop software (Adobe) and analyzed using ImageJ software. For observation of antheridiophers, samples were frozen in liquid nitrogen and captured using a VHX-D500 microscope (KEYENCE, Osaka, Japan).

## Results

### Liverwort *M.polymorpha *has a single DYRKP ortholog

**Fig. 1:**
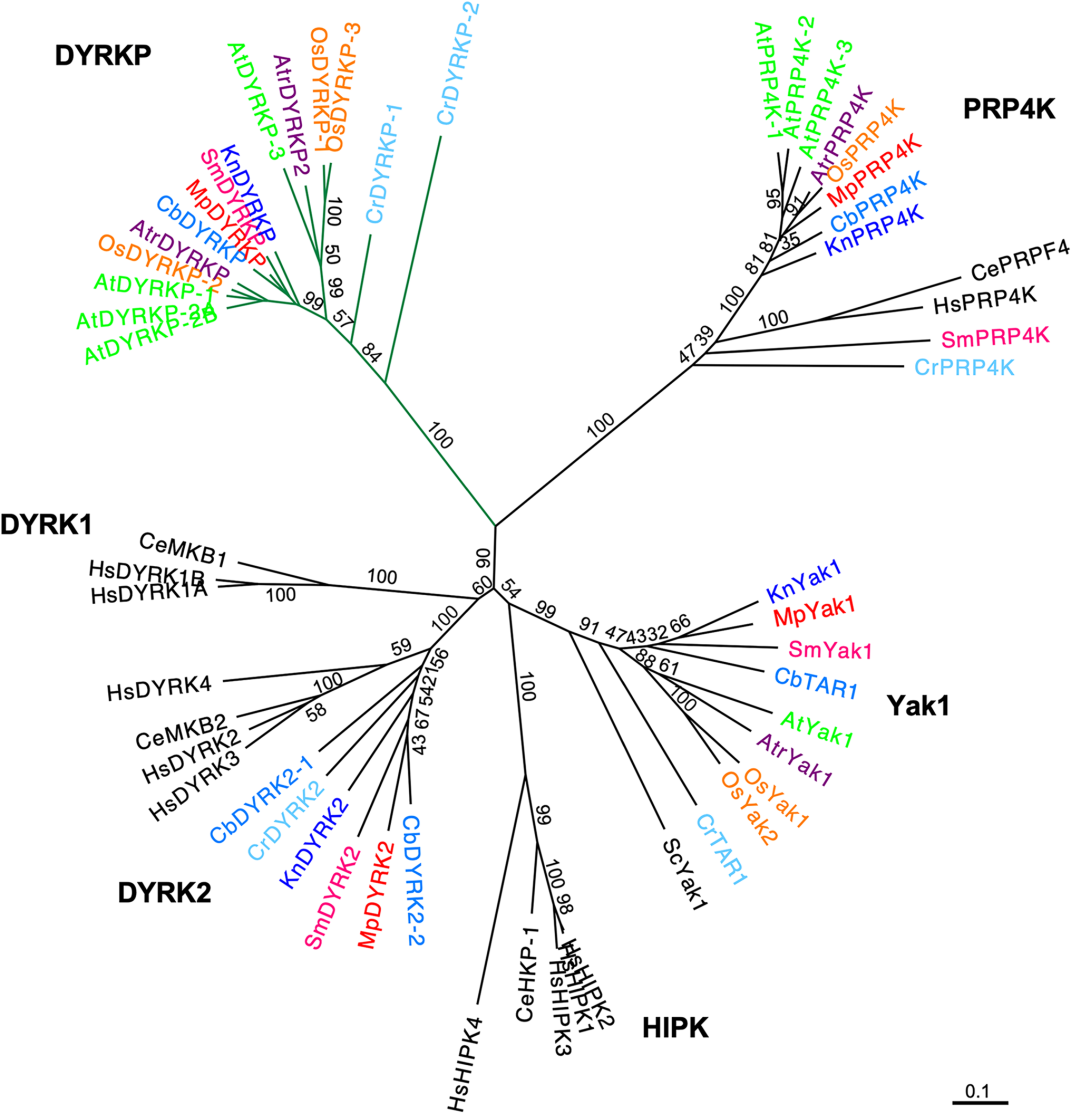
Phylogenic tree of dual-specificity tyrosine phosphorylation-regulated kinases (DYRKs). This phylogenic tree was constructed by comparing the amino acid sequences of the kinase domains from different plant species (*Arabidopsis thaliana* [At], *Oryza sativa* [Os], *Amborella trichopoda* [Atr], *Selaginella moellendorffii* [Sm], *Marchantia polymorpha* [Mp], *Chara braunii* [Cb], *Klebsormidium nitens* [Kn], and *Chlamydomonas reinhardtii* [Cr]), animals (*Caenorhabditis elegans* [Ce] and *Homo sapiens* [Hs]), and yeast (*Saccharomyces cerevisiae* [Sc]). Plant DYRKs are indicated with different colors for each plant species. The plant specific DYRKP subgroup node is green. The tree was generated using MEGA X with the maximum likelihood algorithm. Bootstrap values (1,000 replicates) are indicated at branch nodes, and the scale bar indicates the number of amino acid substitutions per site

**Fig. 2:**
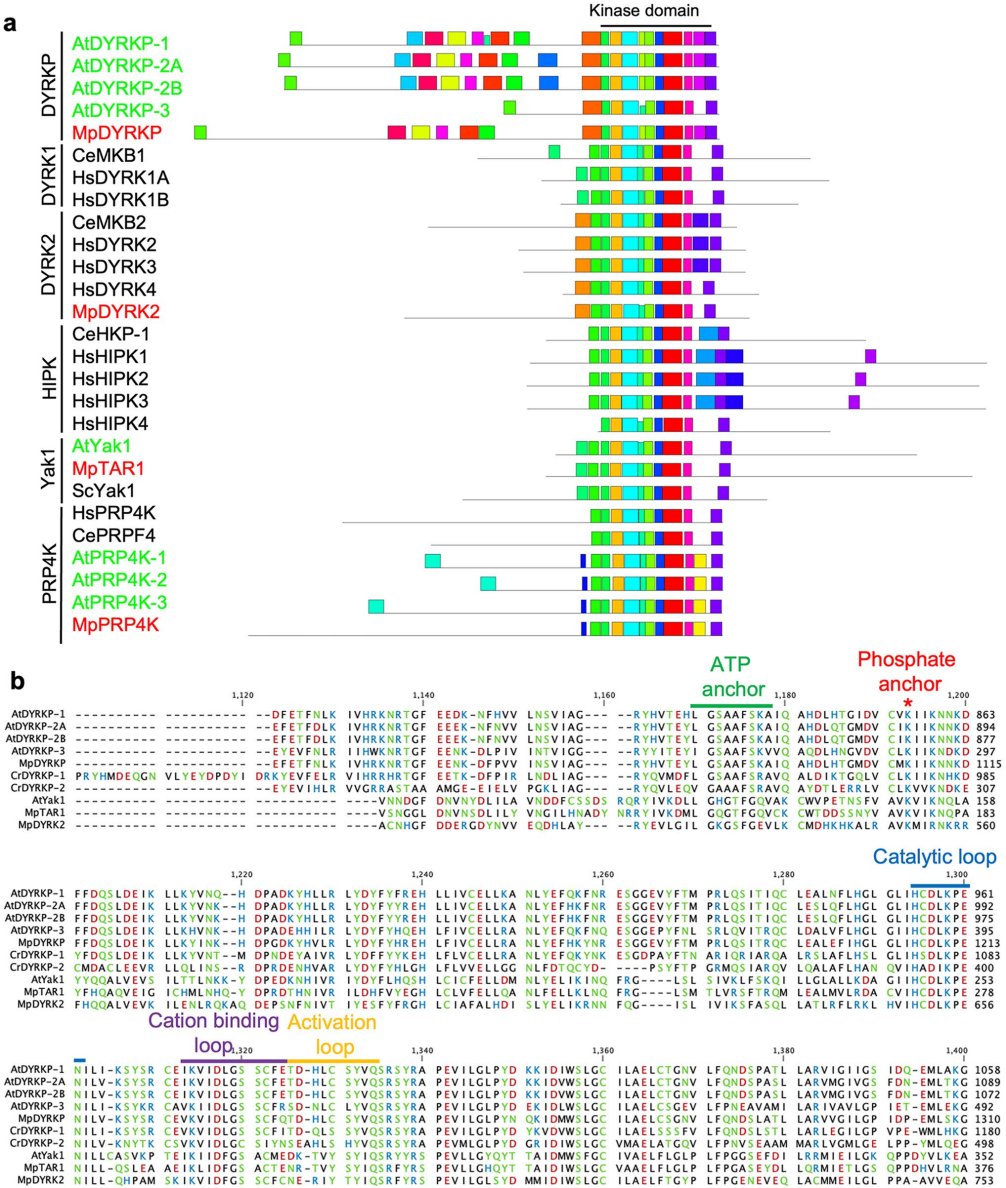
DYRK amino acid sequence comparison amongst different species. **a** Sequence motif among DYRKs. Comparison analysis of DYRKs in *A. thaliana* (At), *M. polymorpha* (Mp), *C. elegans* (Ce), and *H. sapiens* (Hs) was preformed using MEME suites. **b** Multiple sequence alignments were constructed using MUSCLE. Alignments for a part of the kinase domain of DYRKPs (AtDYRKPs, MpDYRKP, CrDYRKPs), YAK1s (AtYak1 and MpTAR1), and DYRK2 (MpDYRK2) are indicated. Residues were colored according to polarity. Important sequence features for kinase activity are indicated

**Fig. 3:**
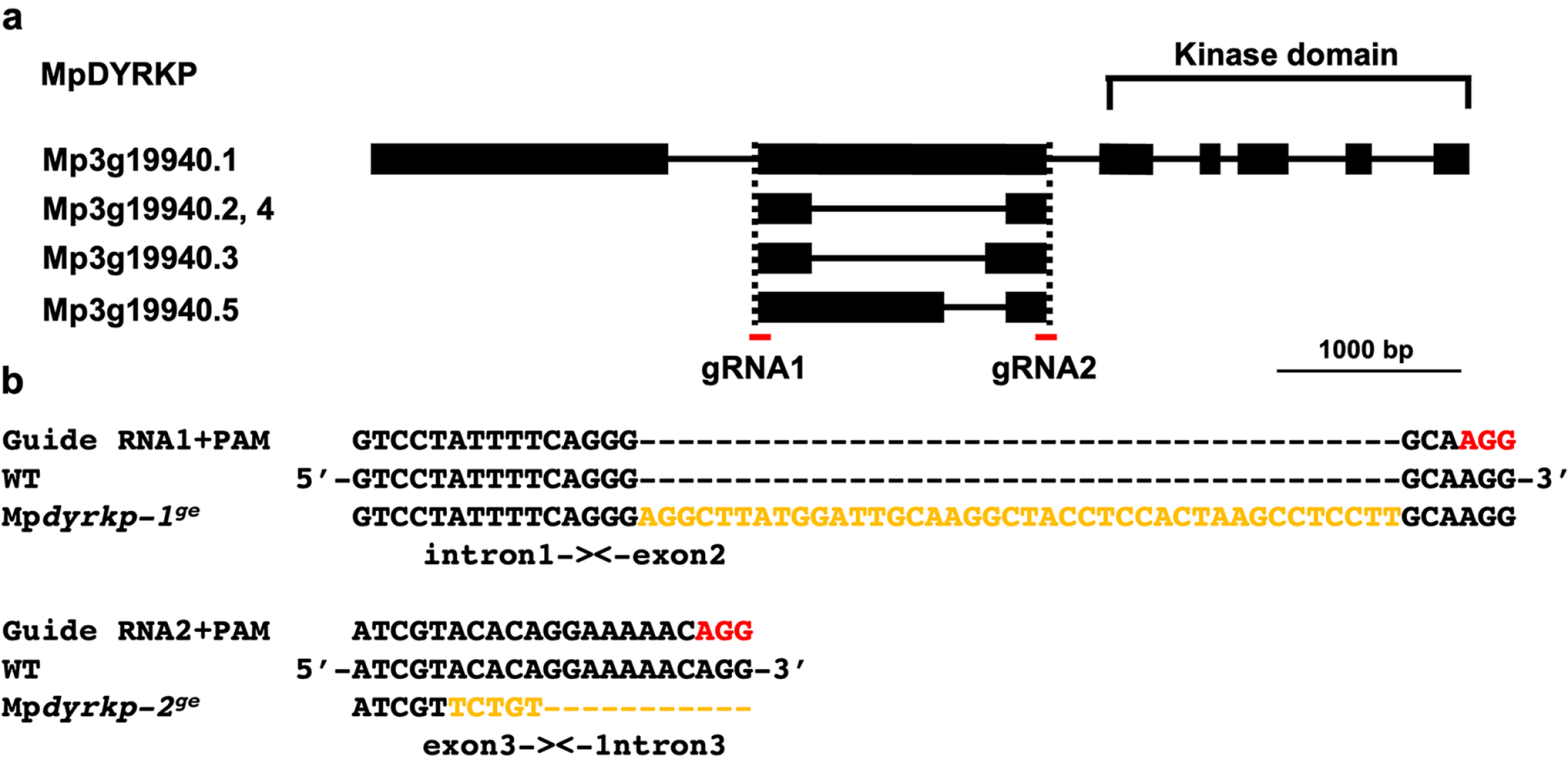
Construction of Mp*dyrkp* genome-edited lines. **a** Genomic structure of the encoding region for the Mp*DYRKP* gene. Red bars indicate the locations of the target sites for the gRNAs. **b** Mutations detected by sequencing analysis. The guide RNA including the PAM sequence (red) and a part of the Mp*DYRKP* sequence in both the wild-type and Mp*dyrkp*^*ge*^ lines are shown. The genome-edited line Mp*dyrkp-1*^*ge*^ has a 41-bp insertion, while Mp*dyrkp-2*^*ge*^ has a 5-bp substitution and a 11-bp deletion (orange)

The plant-specific subgroup in the DYRK family, known as the DYRKP subgroup, widely exists from algae to seed plants (Kajikawa et al. 2015; Schulz-Raffelt et al. 2016). Based on a BLAST search and phylogenetic analysis, we identified a single member in the DYRKP subgroup, Mp*DYRKP* (Mp3g19940), in the genome of the basal land plant *M. polymorpha* (MarpolBase genome database [v5.1: http://marchantia.info]; Fig. 1; Montgomery et al. 2020). MpDYRKP has a highly conserved S/T protein kinase domain in the C-terminal region, which is homologous to other DYRKP orthologs (Fig. 2a). MpDYRKP possesses motifs and amino acids that are important for kinase activity, such as a catalytic loop and a lysine residue, which is a phosphate-anchor to support ATP interactions (Fig. 2b; Aranda et al. 2011). However, although the YxY sequence motif is conserved in the activation loop of the DYRK, DYRK2, and Yak1 subgroups, in the DYRKP subgroup it is conserved as C/SxY (Fig. 2b). Transcriptomic analysis of the *M. polymorpha* genome estimated five alternative splicing variants as MpDYRKP gene models (Fig. 3a; Bowman et al. 2017; Montgomery et al. 2020). All alternative splicing variants of MpDYRKP differed within the N-terminal region (Fig. 3a). The N-terminal region of MpDYRKP has no known domains or motifs, as judged by Pfam (http://pfam.xfam.org). In contrast, the identification of *de novo* sequence motifs by MEME suites (https://meme-suite.org/meme/index.html) revealed that there are highly conserved N-terminal region sequence motifs among the DYRKP subgroup (Fig. 2a).

### Morphology of the genome-edited Mp*dyrkp* mutants

To investigate the roles of MpDYRKP in *M. polymorpha*, Mp*dyrkp* genome-edited lines were created using the CRISPR/Cas9 system (Sugano et al. 2018). We designed two target sites to obtain independent alleles for deficient mutants, and consequently isolated two genome-edited lines, Mp*dyrkp-1*^*ge*^ and Mp*dyrkp-2*^*ge*^ (Fig. 3b). Mp*dyrkp-1*^*ge*^, which is constructed using gRNA1, has a 41-bp insertion, while Mp*dyrkp-2*^*ge*^, which is constructed using gRNA2, has a 5-bp substitution and an 11-bp deletion (Fig. 3b).

**Fig. 4:**
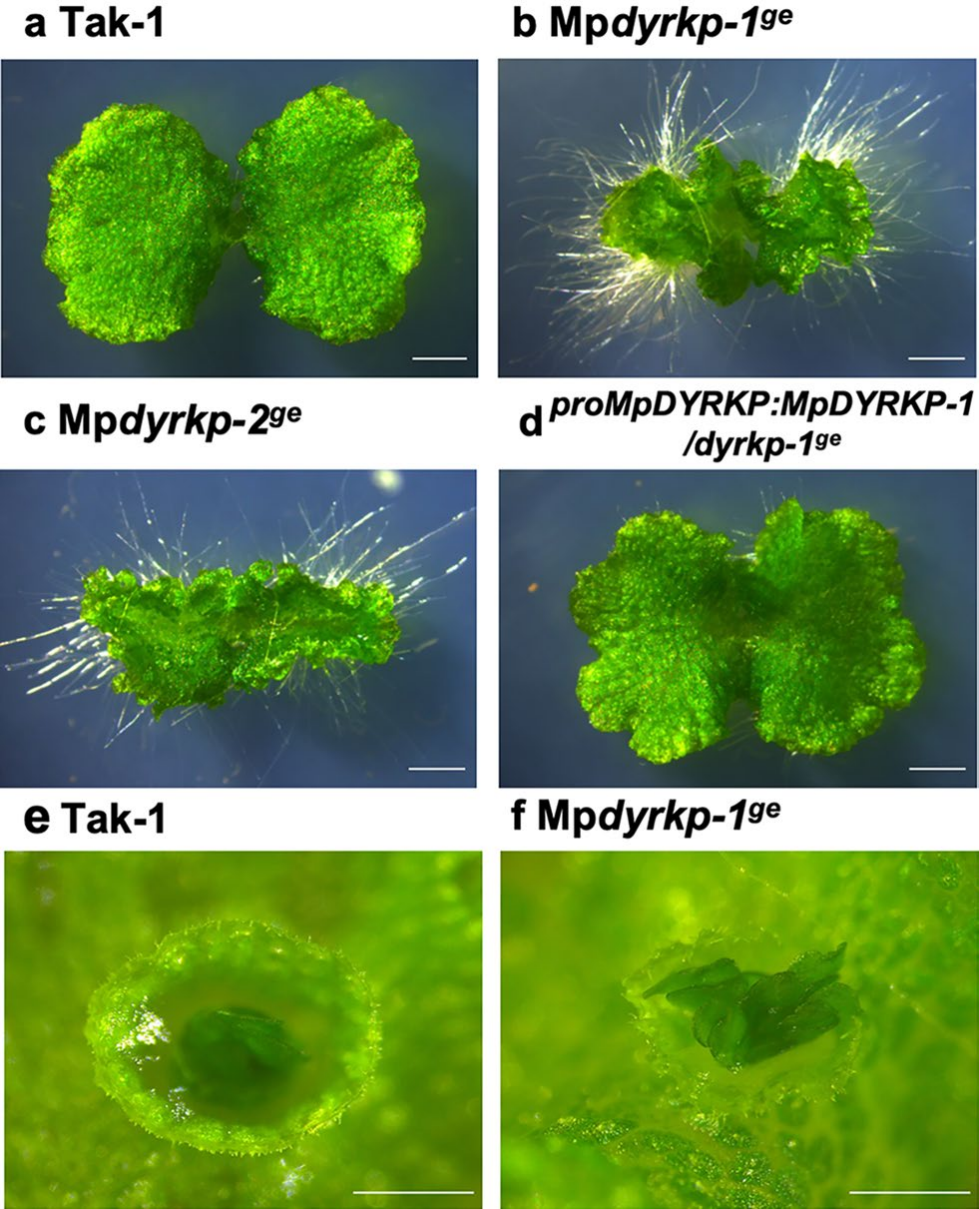
Morphology of Mp*dyrkp* mutants. **a–d** 12-day-old gemmalings of the wild-type line Tak-1 (**a**); the Mp*dyrkp* genome-edited lines Mp*dyrkp-1*^*ge*^ (**b**) and Mp*dyrkp-2*^*ge*^ (**c**); and the complementation line *pro*Mp*DYRKP*:Mp*DYRKP*-1/Mp*dyrkp-1*^*ge*^ (**d**). **e-f** Gemma cup of Tak-1 (**e**) and Mp*dyrkp-1*^*ge*^ (**f**) cultured for 16 days. Scale bars: 2 mm (**a–d**), 1 mm (**e, f**)

**Fig. 5:**
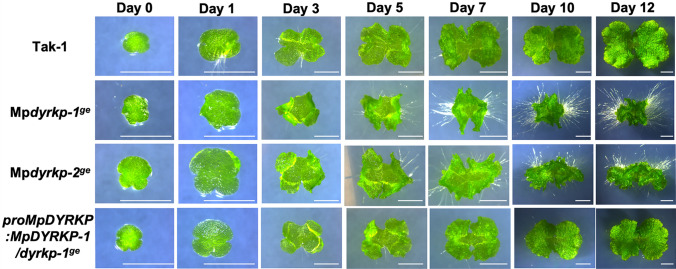
Time-course observation of Mp*dyrkp* mutants. The wild-type line Tak-1; the Mp*dyrkp* genome-edited lines Mp*dyrkp-1*^*ge*^ and Mp*dyrkp-2*^*ge*^; and the complementation line; *pro*Mp*DYRKP*:Mp*DYRKP*-1/Mp*dyrkp-1*^*ge*^ were observed at the indicated time points during growth from the gemma. Scale bars: 2 mm

**Fig. 6:**
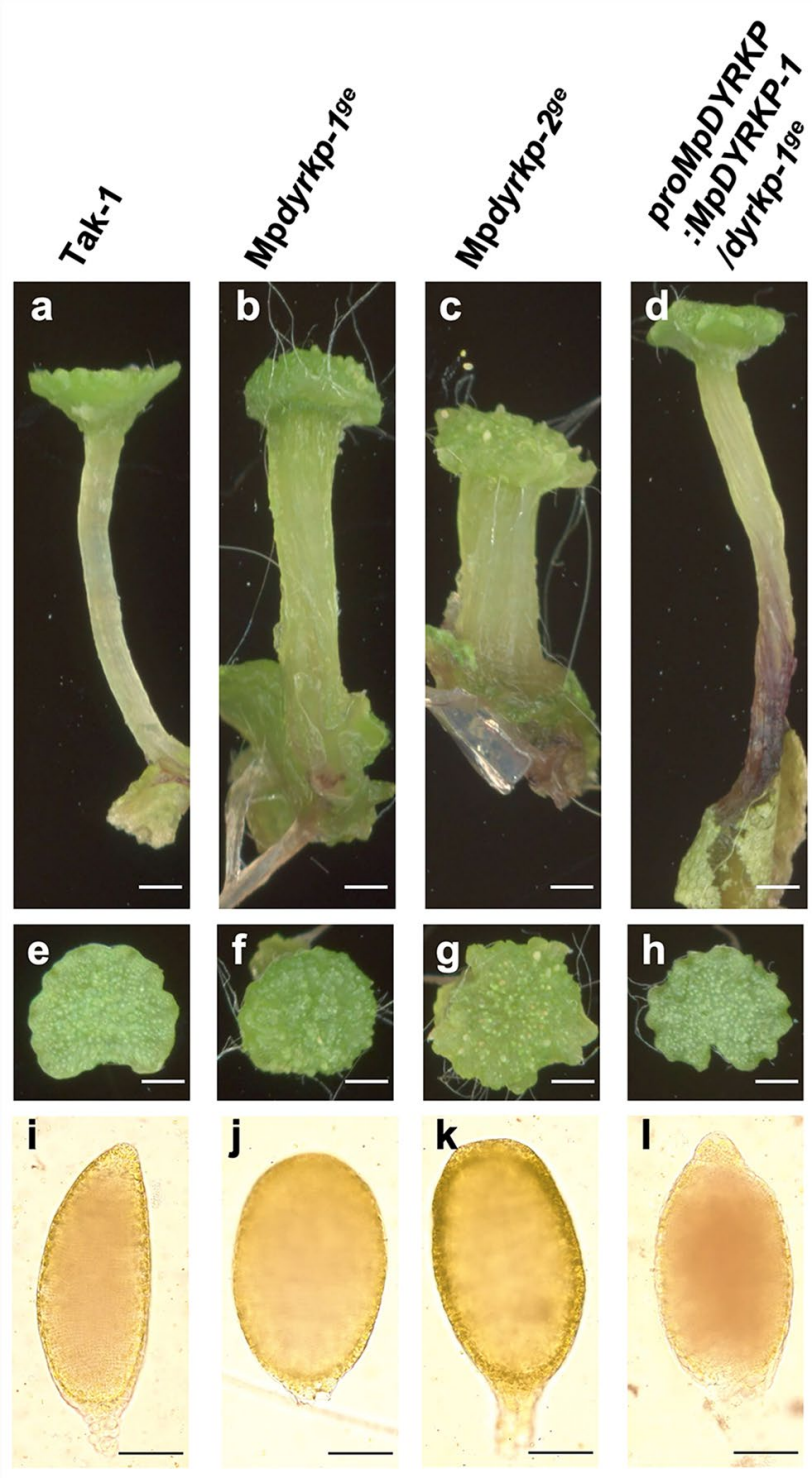
Morphology of the male reproductive organs of Mp*dyrkp* mutants. Side (**a-d**) and top (**c**-**d**) views of antheridiophores of Tak-1 (**a,** **e**), Mp*dyrkp-1*^*ge*^ (**b,** **f**), Mp*dyrkp-2*^*ge*^ (**c,** **g**), and *pro*Mp*DYRKP*:Mp*DYRKP*-1/Mp*dyrkp-1*^*ge*^ (**d,** **h**). Antheridia of Tak-1 (**i**), Mp*dyrkp-1*^*ge*^ (**j**), Mp*dyrkp-2*^*ge*^ (**k**), and *pro*Mp*DYRKP*:Mp*DYRKP*-1/Mp*dyrkp-1*^*ge*^ (**l**).Scale bars: 1 cm (**a–h)**, 100 μm **(i–l)**

In 12-day-old plants after gemma germination, both genome-edited lines Mp*dyrkp-1*^*ge*^ and Mp*dyrkp-2*^*ge*^ exhibited growth defects with abnormal thallus morphologies when compared with the wild-type line Tak-1 (Fig. 4). The thalli of both Mp*dyrkp* mutants were shrunken and less flattened, and their margins were waved (Fig. 4). To confirm the contributions of MpDYRKP to the morphological features of the Mp*dyrkp* mutants, we constructed a complementation line, *pro*Mp*DYRKP*:Mp*DYRKP*-1/Mp*dyrkp-1*^*ge*^. The thallus morphology of this complementation line was restored to that of the wild-type, demonstrating that the abnormal morphologies of the Mp*dyrkp* gene-edited lines were caused by the loss-of-function of MpDYRKP (Fig. 4). This complementation line uses a 5 kbp region upstream of the start codon as an estimated promoter region and its coding sequence. This promoter region was sufficient to express Mp*DYRKP* as a morphological regulator (Fig. 4). To examine in detail the differences between the wild-type and mutant lines, we traced the development of gemmalings for 12 days (Fig. 5). After day 3, both Mp*dyrkp* mutants showed vertical growth in their thallus margin when compared with the wild-type and complementation lines (Fig. 5), indicating that the morphological defects in Mp*dyrkp* are expressed in the early developmental stages of the thallus. *M. polymorpha* propagates asexually via gemmae formation (Kato et al. 2020; Shimamura 2016). Gemmae are formed at the bottom of the gemma cup, which is a cup-like organ that develops on the middle rib of the thallus. The shape of the gemma cup in Mp*dyrkp-1*^*ge*^ was shallow and distorted when compared to the wild-type (Fig. 4). In addition to these morphologies during the vegetative growth phase, morphological differences in the reproductive organs were observed. The male reproductive organs, antheridiophores, of Mp*dyrkp-1*^*ge*^ and Mp*dyrkp-2*^*ge*^ showed abnormal morphology with less flattening and waved margins, similar to its thallus morphology (Fig. 6). In addition, the stalks of antheridiophore of Mp*dyrkp-1*^*ge*^ and Mp*dyrkp-2*^*ge*^ were thicker than Tak-1 and *pro*Mp*DYRKP*:Mp*DYRKP*-1/Mp*dyrkp-1*^*ge*^ (Fig. 6). These results suggest that MpDYRKP plays an important role in tissue morphogenesis in both the vegetative and reproductive phases. On the other hand, antheridia showed normal development in Mp*dyrkp-1*^*ge*^ and Mp*dyrkp-2*^*ge*^ (Fig. 6i–l).

### Subcellular localization pattern of MpDYRKP

**Fig. 7:**
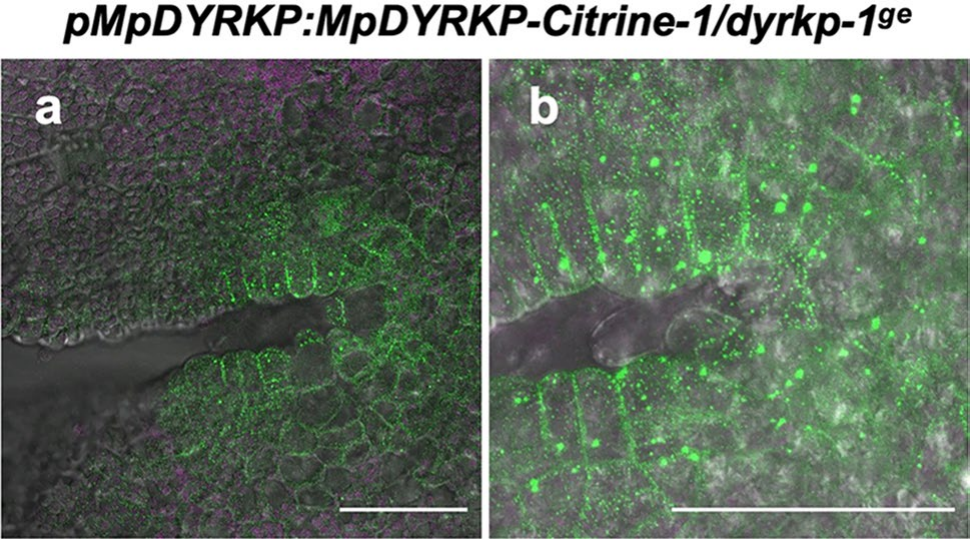
Subcellular localization of MpDYRKP-Citrine protein. **a** and **b** Subcellular localization of MpDYRKP-Citrine in 1-day-old gemmalings of *pro*Mp*DYRKP*:Mp*DYRKP-Citrine-1*/Mp*dyrkp-1*^*ge*^ transgenic plant was visualized with DIC image Citrine signals are shown in green and autofluorescence in magenta. **b** Is a magnified image from around the apical notch in **a**. Scale bars: 50 μm

To analyze the gene expression pattern of Mp*DYRKP*, we re-analyzed the published RNA-seq data sets from various tissues containing thallus, reproductive tissues (antheridiophore, antheridium, and archegoniophore), and developing spores (Bowman et al. 2017; Higo et al. 2016). Mp*DYRKP* was broadly expressed in all the analyzed tissues (Fig. S1). For detailed expression pattern and protein localization analysis, we constructed a reporter line, *pro*Mp*DYRKP*:Mp*DYRKP-Citrine-1*/Mp*dyrkp-1*^*ge*^, which used a 5 kbp region upstream from the start codon of Mp*DYRKP* as a promoter, a process similar to that used for the complementation line. Our observations of this reporter line revealed that MpDYRKP-Citrine was strongly detected around the apical notch (Fig. 7). Moreover, at the subcellular level, MpDYRKP-Citrine was observed as a diffuse punctate structure in the cytosol (Fig. 7). This subcellular localization pattern is different from that of *A. thaliana* DYRKP-2 A, which is uniformly located in the cytosol with no punctate structure (Iwabuchi et al. 2019).

### Cell proliferation activity of Mp*dyrkp* mutants

**Fig. 8:**
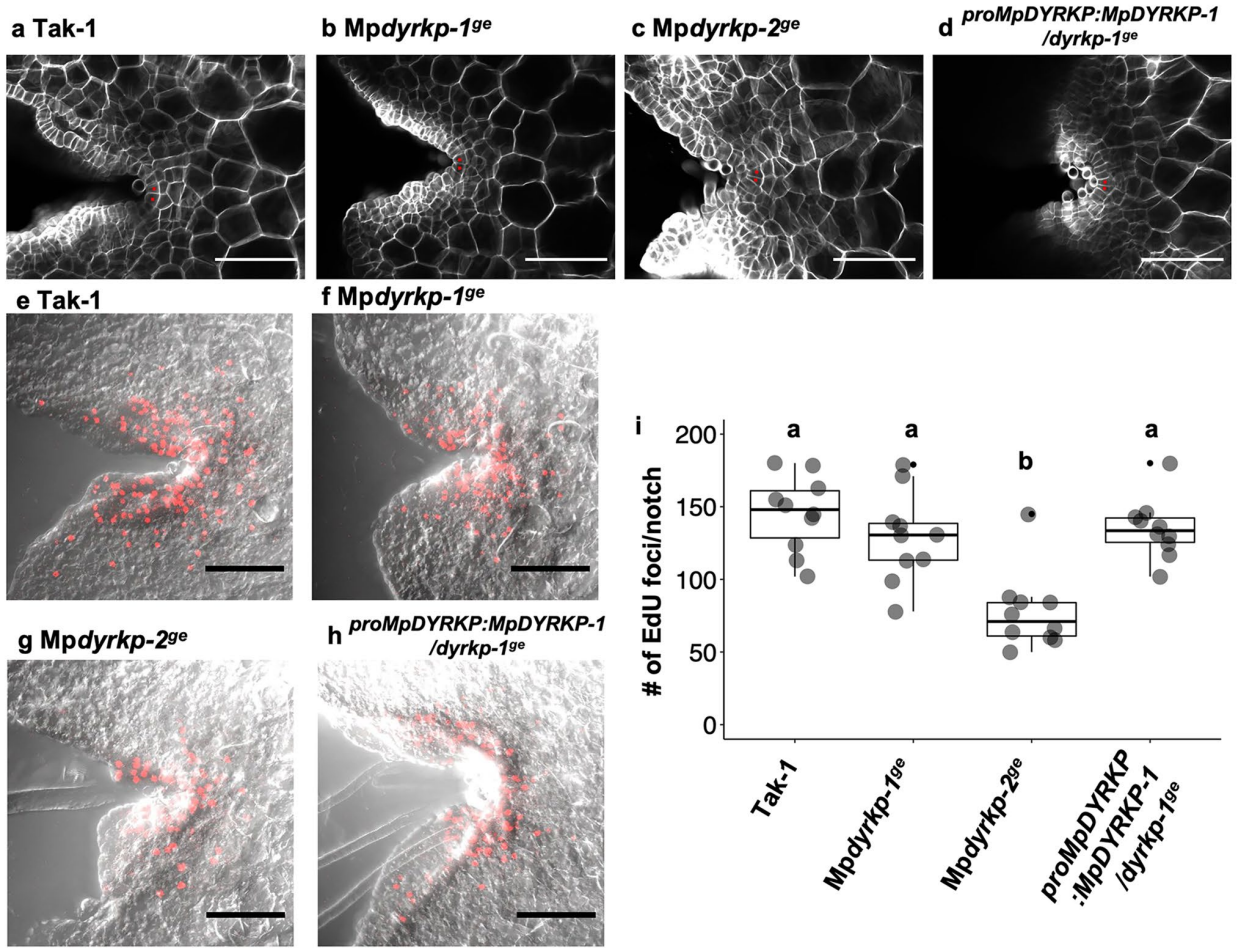
Cell proliferation activity in Mp*dyrkp* mutants. **a–d** Conforcal images of the apical notches in the Tak-1 (**a**), Mp*dyrkp-1*^*ge*^ (**b**), Mp*dyrkp-2*^*ge*^ (**c**), and *pro*Mp*DYRKP*:Mp*DYRKP-1*/Mp*dyrkp-1*^*ge*^ (**d**). Plants cultured for 3 days after gemma germination were treated with ClearSee solution. Cell walls stained by SCRI Renaissance 2200 are shown in white. Red dots indicate apical and/or subapical cells. **e–h** S-phase nuclei visualized by EdU in the Tak-1 (**e**), Mp*dyrkp-1*^*ge*^ (**f**), Mp*dyrkp-2*^*ge*^ (**g**), and *pro*Mp*DYRKP*:Mp*DYRKP-1*/Mp*dyrkp-1*^*ge*^ (**h**). Plants cultured for 3 days after gemma germination were treated with EdU-containing liquid medium for 1 h. Z-series fluorescence images were obtained. Maximum projection images are shown. EdU-containing nuclei are shown in red. **i** The numbers of EdU-containing nuclei are shown in box-and-whisker plots. The upper and lower limits indicate the first and third interquartile ranges, respectively, and the middle bar indicates the median. Whiskers indicate × 1.5 the interquartile ranges. The black dot indicates an outlier. Gray dots indicate each sample (*n* = 10 apical notches). Different lowercase letters indicate significant differences (*P* < 0.05; Tukey–Kramer test). Scale bars: 100 μm (**a–d**), 50 μm (**e–h**)

During thallus development, cell proliferation mainly occurrs in the apical notches (Shimamura 2016). Thus, the apical notch is important for thallus morphogenesis. Although the Mp*dyrkp* gene-edited lines exhibited abnormal thallus morphology (Figs. 4 and 5), presense of the apical or subapical cells in the apical notch region were recognized in both Mp*dyrkp-1*^*ge*^ and Mp*dyrkp-2*^*ge*^ as well as Tak-1 and *pro*Mp*DYRKP*:Mp*DYRKP*-1/Mp*dyrkp-1*^*ge*^ (Fig. 8a–d). Next, we measured the number of S-phase cells visualized by EdU staining in the apical notches of 3-day-old plants to assess the cell proliferation activity. The number of EdU signals in the apical notches of Mp*dyrkp-1*^*ge*^ was comparable to that of Tak-1 and *pro*Mp*DYRKP*:Mp*DYRKP*-1/Mp*dyrkp-1*^*ge*^ (Fig. 8e–i). These results suggest that both apical notch formation and cell proliferation activity are not casual factors for the morphological abonormalities in the Mp*dyrkp* mutants. On the other hand, Mp*dyrkp-2*^*ge*^ exhibited smaller number of EdU signals in their apical notches compared with Tak-1 (Fig. 8e–i), suggesting that Mp*dyrkp-2*^*ge*^ may have additional mutations relate to the cell proliferation activity.

### Epidermal cell morphology of Mp*dyrkp* mutants

**Fig. 9:**
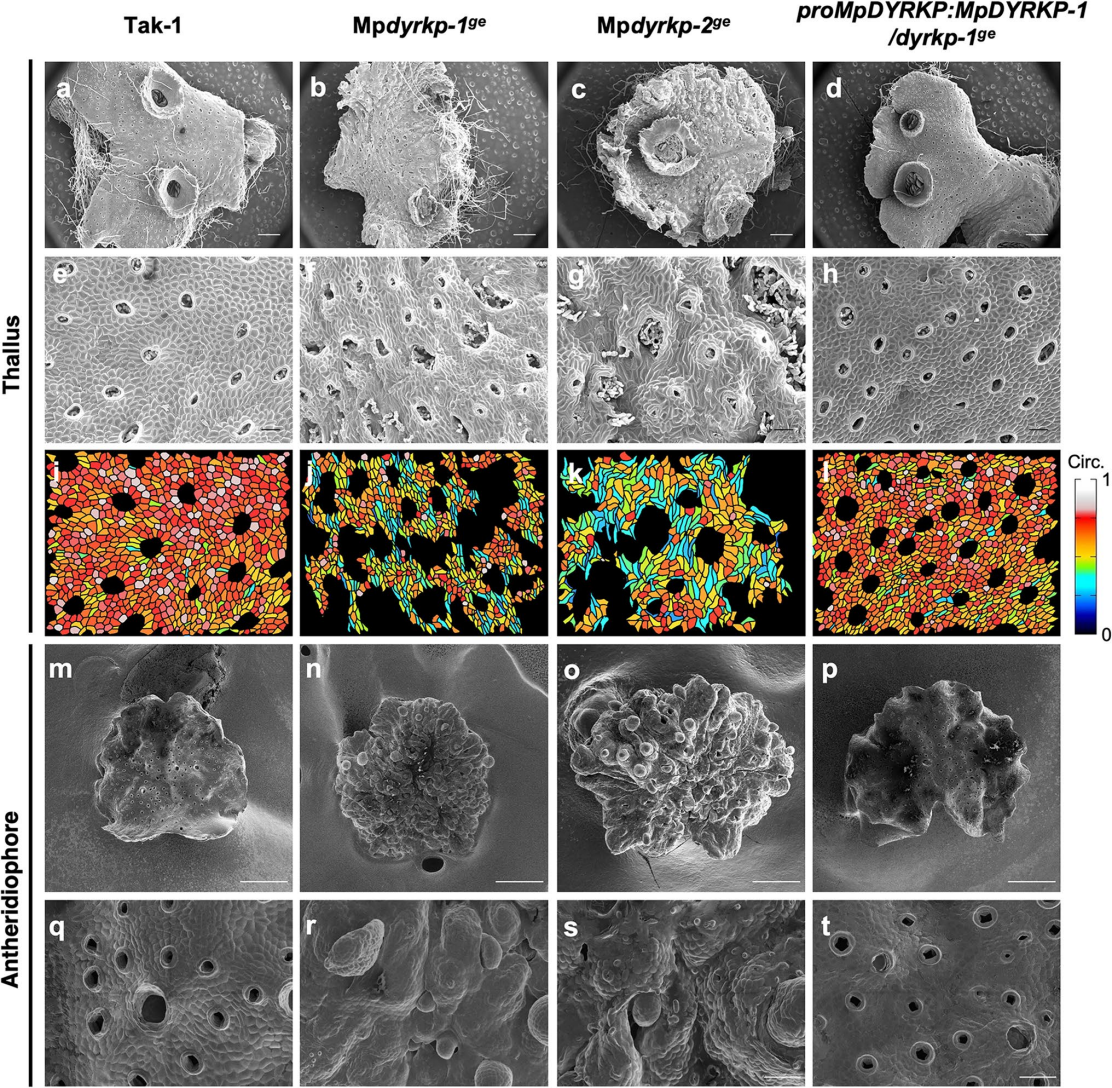
Epidermal morphology in Mp*dyrkp* mutants. **a–h** Scanning electron microscopy images of thalli of Tak-1 (**a,** **e**), Mp*dyrkp-1*^*ge*^ (**b,** **f**), Mp*dyrkp-2*^*ge*^ (**c,** **g**), and *pro*Mp*DYRKP*:Mp*DYRKP-1*/Mp*dyrkp-1*^*ge*^ (**d,** **h**). **i–l** Epidermal-cell circularity shown in scanning electron microscopy images (**e–h**) were measured using Image J. **m-t** Scanning electron microscopy images of antheridial receptacles of Tak-1 (**m** and **q**), Mp*dyrkp-1*^*ge*^ (**n** and **r**), Mp*dyrkp-2*^*ge*^ (**o,** **s**), and *pro*Mp*DYRKP*:Mp*DYRKP-1*/Mp*dyrkp-1*^*ge*^ (**p,** **t**). Scale bars: 1 mm (**a–d, m–p**), 100 μm (**e–h, q–t**)

The morphological defects in the Mp*dyrkp* genome-edited lines, suggests that MpDYRKP acts as a morphological regulator (Figs. 4 and 5). As tissue morphogenesis is an accumulation of’ cellular morphogenesis, we observed the genome-edited lines, Mp*dyrkp-1*^*ge*^ and Mp*dyrkp-2*^*ge*^, using scanning electron microscopy. In contrast to the smooth epidermal surface of Tak-1, the epidermal surfaces of both Mp*dyrkp-1*^*ge*^ and Mp*dyrkp-2*^*ge*^ appeared uneven and disordered (Fig. 9a–h). In addition, the shape and size of the air pores in these genome-edited lines were uneven (Fig. 9a–h). Similar trends were observed in the surfaces of antheridial receptacles of both Mp*dyrkp-1*^*ge*^ and Mp*dyrkp-2*^*ge*^ (Fig. 9m–t). Moreover, we found that both Mp*dyrkp-1*^*ge*^ and Mp*dyrkp-2*^*ge*^ exhibited a tendency for lower circularity in the epidermal cells of thalli than Tak-1 and the complementation line (Fig. 9i–l). These results suggest that MpDYRKP contributes to thallus morphogenesis, at least in part, via the regulation of epidermal cell shape.

## Discussion

### MpDYRKP is involved in the regulation of tissue morphogenesis

Mp*dyrkp* genome-edited lines of the model basal land plant *M. polymorpha*, were shown here to exhibit morphological defects in tissue morphogenesis in both the vegetative and reproductive phases (Figs. 4, 5 and 6). This is the first report regarding the phenotype of a loss-of-function mutant of a DYRKP ortholog in bryophytes. At the cellular level, the epidermal cells were found to have an altered shape with lower circularity in the Mp*dyrkp* mutants when compared with the wild type (Fig. 9). According to abnormal cell shapes, the epidermal surface of the Mp*dyrkp* mutants was uneven and disordered (Fig. 9). The disorder in the shape and alignment of the epidermal cells of the Mp*dyrkp* mutants may account for the morphological disruption of orderly tissues such as the thalli, air pores, and gemma cups (Figs. 4 and 9). These results suggest that MpDYRKP regulates cell morphology to enable well-ordered development in *M. polymorpha*. Generally, cytoskeletons, including microtubules and actin filaments, play important roles in the regulation and determination of plant cell shape (Smith and Oppenheimer 2005). It has been suggested that *A. thaliana* DYRKPs (AtDYRKPs) are involved in nuclear positioning via the regulation of actin filament alignment (Iwabuchi et al. 2019). Moreover, in humans, a member of the DYRK1 subgroup, DYRK1A, induced the destabilization of microtubules via the hyperphosphorylation of tau proteins, which is a causal agent of Alzheimer’s disease (Ryoo et al. 2007; Woods et al. 2001). Thus, it is important to analyze the molecular functions and identify the interacting proteins of MpDYRKP to understand the underlying mechanisms of tissue morphogenesis in *M. polymorpha.*

### Relationship between DYRKPs and AN

In *A. thaliana*, DYRKPs were found to interact with AN proteins in the yeast two-hybrid screens (Bhasin and Hülskamp 2017: ANGSTIFOLIA INTERACTING KINASE [AIK1] corresponds to AtDYRKP-1), and the co-immunoprecipitation assay using transgenic plants expressing AN-GFP (Iwabuchi et al. 2019). Mutants deficient in AN exhibited multiple morphological phenotypes, such as narrow and thicker leaves, twisted fruits and petals, and the premature opening of flowers (Bai et al. 2010, 2013; Rédei 1962; Tsuge et al. 1996; Tsukaya et al. 1994). In *M. polymorpha*, the knockout mutants of the AN ortholog, Mp*an*, showed abnormal twisted thalli and suppressed thallus growth, indicating that MpAN is also involved in the regulation of tissue morphogenesis (Furuya et al. 2018). Moreover, in both *A. thaliana* and *M. polymorpha*, the loss of function of AN results in an abnormal arrangement of the cortical microtubules (Furuya et al. 2018; Kim et al. 2002). The phenotypes between Mp*dyrkp* and Mp*an* mutants were partially shared in the context of abnormal morphologies with less flattening of the thalli and antheridial receptacles (Figs. 4, 5 and 6). However, it is not easy to compare the phenotypic similarities between the Mp*dyrkp* and Mp*an* mutants due to their morphological complexity. In terms of epidermal cell shapes, Mp*dyrkp* showed lower circularity than the wild type under normal growth conditions (Fig. 9), whereas Mp*an* showed higher circularity only under weak blue light conditions (Furuya et al. 2018). These results indicate that the effects of the Mp*dyrkp* mutation are more severe and opposing to those of the Mp*an* mutation. The protein localization of MpDYRKP-Citrine was observed as a diffuse punctate structure in the cytosol (Fig. 7). This localization pattern is different from that of GFP-DYRKP-2 A in *A. thaliana*, which is uniformly located in the cytosol with no punctate structure (Iwabuchi et al. 2019). Interestingly, the diffuse punctate structures observed in MpDYRKP-Citrine transgenic plants were similar to the AN protein localization pattern observed in both *M. polymorpha* and *A. thaliana* (Bhasin and Hülskamp 2017; Furuya et al. 2018). Further studies of the molecular and genetic interactions between DYRKP and AN in *M. polymorpha* will provide clues to uncover their functional mechanisms in tissue and cell morphogenesis.

## Conclusions

In this study, we revealed that MpDYRKP is involved in the regulation of cell shape to enable the formation of orderly tissue morphologies in *M. polymorpha*. However, the molecular function, target substrates, and physiological roles of MpDYRKP and other DYRKPs remain unknown. Comparison of the DYRKPs revealed that features of the amino acid sequences of MpDYRKP are conserved across a broad range of plant species. Thus, the molecular functions of MpDYRKP, as a morphological regulator in *M. polymorpha*, may have been conserved during plant evolution.

## Supplementary Information

Below is the link to the electronic supplementary material.

Supplementary file1 (DOCX 707 KB)
