# Supplementary material for: A plant-specific DYRK kinase DYRKP coordinates cell morphology in Marchantia polymorpha
Source: J Plant Res. 2021 Sep 21;134(6):1265–77. doi: 10.1007/s10265-021-01345-w (PMC8514375; doi:10.1007/s10265-021-01345-w)
Supplement: Supplementary file 1 — Supplementary file1 (DOCX 707 KB) [file 10265_2021_1345_MOESM1_ESM.docx]

**Supplementary Information**

**A plant-specific DYRK kinase DYRKP coordinates cell morphology in *Marchantia polymorpha***

**Journal of Plant Research**

Tomoyuki Furuya^1,2^, Haruka Shinkawa^3,4^, Masataka Kajikawa^3,5^, Ryuichi Nishihama^3,6^, Takayuki Kohchi^3^, Hideya Fukuzawa^3^, Hirokazu Tsukaya^2^*

1. Graduate School of Science, Kobe University, Kobe 657-8501, Japan

2. Graduate School of Science, The University of Tokyo, Tokyo 113- 0033, Japan

3. Graduate School of Biostudies, Kyoto University, Kyoto 606-8502, Japan

4. Present Address: Research Institute for Bioresources and Biotechnology, Ishikawa Prefectural University, Ishikawa 921-8836, Japan

5. Present Address: Faculty of Biology-Oriented Science and Technology, Kindai University, Wakayama 649-6493, Japan

6. Present Address: Faculty of Science and Technology, Tokyo University of Science, Chiba 278-8510, Japan.

*** Corresponding author:** [**tsukaya@bs.s.u-tokyo.ac.jp**](mailto:tsukaya@bs.s.u-tokyo.ac.jp)


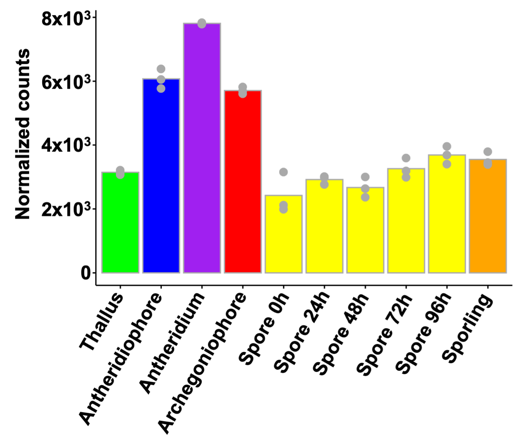


**Fig. S1 Gene expression pattern of Mp*DYRKP*.** Expression levels of Mp*DYRKP* gene in various organs from the published RNA-seq data sets (Higo et al., 2016 and Bowman et al., 2017) are indicated. *Marchantia* genome (v5.1r2) were used as references for HISAT2 (v2.2.0) mapping. Read counts were calculated and normalized by StringTie (2.1.3b) and edgeR (v5.3.2), respectively. Gray dots indicate value in each replicate.
